# p62: Friend or Foe? Evidences for *OncoJanus* and *NeuroJanus* Roles

**DOI:** 10.3390/ijms21145029

**Published:** 2020-07-16

**Authors:** Sonia Emanuele, Marianna Lauricella, Antonella D’Anneo, Daniela Carlisi, Anna De Blasio, Diana Di Liberto, Michela Giuliano

**Affiliations:** 1 Department of Biomedicine, Neurosciences and Advanced Diagnostics (BIND), University of Palermo, Via del Vespro 129, 90127 Palermo, Italy; marianna.lauricella@unipa.it (M.L.); daniela.carlisi@unipa.it (D.C.); diana.diliberto@unipa.it (D.D.L.); 2 Department of Biological, Chemical and Pharmaceutical Sciences and Technologies (STEBICEF), Laboratory of Biochemistry, University of Palermo, Via del Vespro 129, 90127 Palermo, Italy; antonella.danneo@unipa.it (A.D.); anna.deblasio@unipa.it (A.D.B.); michela.giuliano@unipa.it (M.G.)

**Keywords:** p62, autophagy, apoptosis, cancer, neurodegenerative diseases

## Abstract

p62 is a versatile protein involved in the delicate balance between cell death and survival, which is fundamental for cell fate decision in the context of both cancer and neurodegenerative diseases. As an autophagy adaptor, p62 recognizes polyubiquitin chains and interacts with LC3, thereby targeting the selected cargo to the autophagosome with consequent autophagic degradation. Beside this function, p62 behaves as an interactive hub in multiple signalling including those mediated by Nrf2, NF-κB, caspase-8, and mTORC1. The protein is thus crucial for the control of oxidative stress, inflammation and cell survival, apoptosis, and metabolic reprogramming, respectively. As a multifunctional protein, p62 falls into the category of those factors that can exert opposite roles in the cells. Chronic p62 accumulation was found in many types of tumors as well as in stress granules present in different forms of neurodegenerative diseases. However, the protein seems to have a Janus behaviour since it may also serve protective functions against tumorigenesis or neurodegeneration. This review describes the diversified roles of p62 through its multiple domains and interactors and specifically focuses on its *oncoJanus* and *neuroJanus* roles.

## 1. Introduction

p62 is a multifunctional protein that was originally identified as a component of the sequestosome, a cytoplasmic structure which serves as a storage place for ubiquitinated proteins. [1]. The p62 encoding gene was then isolated and termed sequestosome 1 (SQSTM1) [2]. The protein was the first adaptor involved in selective autophagy identified in mammals [3]. Autophagy is a widely described physiological process of recycling cell components through autophagosome formation and lysosomal cargo digestion. The autophagic process requires a number of gene products named Atg proteins [4] and satisfies two major cellular requirements: response to metabolic stress due to nutrient deprivation and detoxification through waste removal. It is thus not surprising that basal autophagy occurs in the cells under normal conditions and that the process highly increases under particular cellular stresses [5,6]. While in “non-selective” autophagy, the autophagosome randomly sequesters cytoplasmic material, in selective autophagy the autophagosome targets specific cargoes and requires selective adaptors [7]. Among selective cargoes, poly-ubiquitin tagged proteins are recognized by specific adaptors or alternatively transmembrane receptor proteins directly bind to cargoes without requiring ubiquitin [8]. Studies on selective protein degradation reveal a crucial role of p62 in autophagic digestion of poly-ubiquitinated protein cargo [9]. During the early steps of autophagosome formation, a dimeric form of p62 is phosphorylated in Ser407 by Atg1/ULK1 kinase, one of the upstream ATG gene products that trigger the autophagic flux. This phosphorylation destabilizes the p62 dimer and renders the protein prone to undergo subsequent phosphorylation by other kinases (casein kinase2 or TANK-binding kinase1) to increase the binding affinity of p62 for ubiquitin chains [10]. It is well known that p62 can preferentially bind to certain ubiquitin chain types and this might represent a subtle level of regulation during selective autophagy. Generally, while Lys48-linked ubiquitin chains are recognized by the 26S proteasome, Lys63-linked ubiquitin chains are also associated with selective autophagy and are preferentially recognized by p62 [11].

Following binding to ubiquitinated proteins, p62 undergoes oligomerization and interacts with the autophagosomal membrane protein LC3, thus delivering the cargo aggregates to the autophagosome [12]. LC3 exerts multiple roles in autophagy including membrane fusion, cargo selection and autophagosome mobilization. LC3/p62 interaction is indispensable for autophagic degradation of polyubiquitinated cargo. Moreover, the same p62 becomes degraded by the autophagosome, which makes the protein a marker to monitor the autophagic flux. Indeed, a precocious increase in p62 cellular levels is usually observed during autophagy execution, and a subsequent decrease, due to autophagic degradation of the protein, occurs later when autophagy is completed [13,14,15,16].

As a multifunctional protein, p62 not only takes part in selective autophagy, but also interacts with many factors that play important roles in determining cell fate. p62 behaves indeed as a signalling hub and can be considered a main regulator of key pathways including the nuclear factor (erythroid-derived 2)-like 2/Kelch-like ECH-associated protein 1 (Nrf2/Keap1) oxidative defense system, NF-kB mediated-inflammation and pro-survival response, mTORC1 related nutrient sensing, and caspase-8- mediated apoptosis [17,18,19,20]. Due to its versatile behaviour and capability of recruiting diverse binding partners to influence many cellular processes, p62 is clearly involved in important diseases including cancer and neurodegenerative disorders [21,22,23]. A common denominator for such a wide range of issues is represented by the balance between cell death and survival, which involves strategic crosstalk between apoptosis and autophagy, metabolic programming, redox balancing, and fine regulation of functional networks determining cell fate. p62 seems to take part in all of these processes and its contribution to their regulation is commonly considered noteworthy, as revealed by a number of studies correlating the factor to both cancer development and neurodegeneration. Intriguingly, p62 behaviour in tumor environment as well as in neuronal circuits is not always univocal. This review aims to highlight the double face of p62, critically discussing the most recent findings in the literature and evidencing the interconnections between pathways involved in cancer development and in neurodegenerative diseases.

## 2. p62 Structure, Post-translational Modifications, and Conformational Changes

Human p62 protein is encoded by SQSTM1 gene, which is located on chromosome 5 and contains 8 exons distributed along 16 kb. Transcriptional activation of SQSTM1 gene can be promoted by various cellular stresses. The SQSTM1 gene promoter indeed contains specific response elements including kB, CLEAR, and ARE, which respond to the respective master gene regulators NF-kB, Mit/TFE, and Nrf2. Specifically, NF-kB can activate p62 under inflammatory or pro-survival responses, Mit/TFE and Nrf2 stimulate p62 expression under metabolic stress and oxidative stress, respectively [24,25,26].

**Figure 1:**
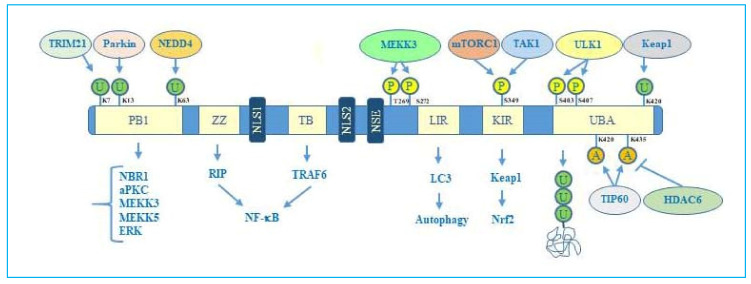
p62 structure, functional domains, post-translational modifications, and interactors. In particular, the figure shows Phox/Bem1p (PB1) domain, which is important for p62 oligomerization, with indicated ubiquitination sites, relative ubiquitinating enzymes (Trim21, parkin, NEDD4) and interactors (NBR1, atypical protein kinase C (aPKC), MEKK3, MEKK5, ERK); Zinc finger ZZ and TRAF bindingTB domains necessary for interaction with receptor interacting protein (RIP) and TRAF6, respectively, to activate NF-κB signalling; nuclear localization sequences (NLS1 and NLS2), and nuclear export sequence (NES) which account for nuclear-cytoplasmic shuttling of p62; LYR and Keap1-interacting region (KIR) domains that interact with LC3 and Keap1 respectively to promote selective autophagy and nuclear factor (erythroid-derived 2)-like 2 (Nrf2)-mediated signalling. Ubiquitin associated (UBA) domain is fundamental for recognition of poly-ubiquitinated cargo during selective autophagy. Post-translational modification sites including phosphorylation, acetylation, and ubiquitination together with the respective regulative enzymes (MEKK3, mTORC1, TAK1, Keap1, Tip60, HDAC6) are also indicated.

Concerning the structure, although human p62 protein is homologous to mouse and rat p62, very different alternative splicing mechanisms have been identified in these models [27,28]. Human p62 exists in two protein isoforms that originate by alternative splicing of three mRNA variants [29]. p62 mRNA variant 1 encodes the full length 440-aa protein. The other two mRNA variants (2 and 3) share the same coding sequence but differ in their 5′UTR regions. Both these variants encode p62 isoform 2, which is 84 amino acids shorter than p62 isoform 1 at the N terminus. While the precise function of this isoform remains unknown, the full length p62 is widely expressed and represents the predominant one. The full length p62 protein structure, including domains and multiple interactors, is reported in Figure 1.

### 2.1. PB1 Domain and p62 Oligomerization

In the N-terminal region Phox/Bem1p (PB1) domain is present, which is responsible for homo-dimerization or formation of p62 oligomeric filaments [30]. While the dimeric form of p62 is substantially inactive with respect to autophagy, polymerization of p62 in a filamentous form, which renders the protein functional, is promoted by the interaction of the protein with the autophagic receptor NBR1 (the neighbor of BRCA1 gene). This factor specifically binds to PB1 domain and cooperates with p62 in targeting poly-ubiquitinated proteins to the autophagosome during selective autophagy [31]. Jacobi at al. have recently elucidated the structural basis of p62 helical filaments formation through the PB1 domain and their role in autophagic cellular cargo uptake [32]. Phase condensation of p62 in non-membrane liquid compartment is crucial for its role of cargo receptor. In this regard, evidence has been provided that p62 “puncta formation”, the process of liquid–liquid phase separation that is needed for p62 oligomerization is enhanced by the cytoplasmic histone H3F3/H3.3 chaperone DAXX [33].

PB1 domain can also interact with other PB1 containing proteins involved in signal transduction including atypical protein kinase C (aPKC), cAMP-degrading phosphodiesterase-4 (PDE4) and MAP kinase pathway members such as MEKK3, MEK5, and ERK [34]. Another p62 binding partner in this region is NEDD4 (neuronal precursor cell expressed developmentally down regulated protein 4). This factor acts as a ubiquitin ligase and is involved in p62 ubiquitination in the PB1 domain facilitating autophagy [35].

### 2.2. The Zinc Finger ZZ Domain, Nuclear Localization, and Export Sequences

The zinc finger domain (ZZ) is located near the PB1 domain and is specifically recognized by receptor interacting protein (RIP). This interaction leads to the activation NF-κB through aPKC- mediated pathway [21]. ZZ is a typical DNA binding domain, however, although two nuclear localization sequences (NLS1 and NLS2) together with a nuclear export sequence (NES) are present, there are not direct evidences that p62 can bind to the DNA.

Regarding the nuclear localization of p62, data present in the literature indicate that p62 knockdown increases chromatin poly-ubiquitination. In this regard, Feng et al. have proposed that p62 is involved in autophagy-regulated DNA repair through inhibition of the E3 ligase RNF168-dependent ubiquitination of histones [36]. Notably, Salmina et al. found nuclear clustering of p62 foci together with the telomere capping protein TRF2 and the DNA damage molecular marker βH2AX in doxorubicin treated breast cancer cells. These authors also found that damaged DNA in the form of telomere fragments was sorted into the cytoplasm in association with p62 [37]. Another interesting study of p62 nuclear localization regards the involvement of the protein in a nuclear form of cell death known as parthanatos, which is dependent on poly (ADPribose) polymerase-1 (PARP-1) [38]. In this study, p62 was shown to be required for nuclear accumulation of aggresome-like structures, resulting in the induction of parthanatos. p62 was also found in association with transcription factors such as TFIIE [39] and TFIIH [40], suggesting a possible role in transcriptional regulation.

### 2.3. The TRAF binding TB Domain

The TRAF binding domain (TB) is located between the NLS1 and NLS2 regions and is specifically recognized by RING finger domain proteins such as tumor necrosis factor (TNF) receptor associated factor 6 (TRAF6). This factor is a ubiquitin E3 ligase, whose activity is critical for activation of NF-κB signalling in response to IL-1 [41]. Therefore, through this domain, p62 also behaves as a significant intermediate of IL-1 signalling pathway to stimulate NF-κB activation during inflammation or pro-survival response. The connection of p62 with NF-κB will be discussed in the next paragraph.

### 2.4. The LC3-Interacting Region (LIR) Domain and Autophagy

LC3-interacting region (LIR) is required for autophagy since it represents the binding site for LC3 to promote the autophagosome formation, thus linking the core autophagy machinery to the target cargo identified by p62. LIR motif, indeed, guides the ubiquitinated cargo captured by p62, through the ubiquitin associated domain (UBA), to Atg8/LC3, which is anchored to the surface of the autophagosomal membrane. Beyond LC3 binding, LIR domain can also interact with other autophagic proteins including beclin and BNIP3 [42,43], representing a fundamental domain for selective autophagy execution.

### 2.5. The Keap1-Interacting Region (KIR) Domain and Nrf2 Signalling Activation

Notably, in addition to degradation of ubiquitinated cargo during selective autophagy, p62 is also involved in the degradation of other substrates such as Kelch-like ECH-associated protein 1 (Keap1), the well-known inhibitor of the oxidative response transcription factor Nuclear factor erythroid-2 related factor 2 (Nrf2). Keap1 binds to a specific motif in p62, called Keap1-interacting region (KIR) which is located next to the LIR domain. KIR is required for p62 to activate Nrf2 through suppression of Keap1 in response to oxidative stress [44]. Phosphorylation of Ser349 in this domain has been shown to be crucial for Keap1 recognition and sequestration [45].

Kageyama et al. have recently identified a p62 splicing variant in mice lacking the KIR domain, this variant produced opposite effects increasing the amount of Keap1, ubiquitination of Nrf2, and consequent suppression of its transcriptional targets [28].

### 2.6. The Ubiquitin Associated (UBA) Domain, Phosphorylation, and Acetylation of p62

The ubiquitin associated domain (UBA) is located next to KIR domain, at the carboxy-terminal region of p62. This domain is a core regulator of the interaction between p62 and the poly-ubiquitinated cargo and is thus necessary for selective autophagy-mediated protein degradation. In an inactive form, homodimerization of UBA domain prevails with consequent impediment of ubiquitinated cargo recognition. Upon autophagic stimuli, phosphorylation by ULK1 kinase in Ser407 present in the UBA domain induces dissociation of the p62 dimer to form a monomer that is subsequently phosphorylated by ULK1 or other kinases such as casein kinase 2 or TANK binding kinase. This modification enhances p62 binding to poly-ubiquitinated proteins. Intriguingly, it has been shown that upon ubiquitin binding p62 acquires liquid like properties since it forms droplets that serve as interactive nodes in the context of selective autophagy. The droplet formation is favoured by the interaction through the PB1 domain of p62 with NBR1, as previously mentioned [31]. Beside p62 regulative phosphorylation within the UBA domain, another important post translational modification is represented by acetylation. In this regard, You et al. have shown that Lys420 and Lys435 in the UBA domain are the main acetylation sites, which respond to TIP60 acetyltransferase and HDAC6 deacetylase [46]. The same authors showed that p62 acetylation occurs under nutrient stress and is required to promote p62 binding to poly-ubiquitinated cargo during selective autophagy.

Overall, the flexible structure of p62 and the fine regulation due to post translational changes makes this protein a versatile interactor and a key factor regulating diverse cellular processes.

## 3. p62 Functional Interplay with Key Factors Regulating Cell Death and Survival

Understanding the molecular basis of highly complex pathologies like cancer and neurodegenerative diseases cannot prescind from an accurate analysis of the delicate balance between cell death and survival within the cells. It is currently recognized that autophagy plays a double role in cancer development as well as in neurodegenerative diseases [47,48]. The process can indeed be promoted as a pro-survival response to particular cellular stresses or it can evolve into a cell death mechanism, namely autophagic cell death, also known as type II programmed cell death [49]. Intriguingly, crosstalk between autophagy and other forms of programmed cell death, including classic apoptosis and/or necroptosis, represents a key point to dissect those networks that establish the cell fate towards either tumorigenesis or neurodegeneration.

p62 has been shown to exert a fundamental role in both autophagy and apoptosis regulation due to its ability to interact with key factors regulating these processes.

Beyond the important role of p62 in binding poly-ubiquitinated cargoes and LC3 recognition during the autophagosome formation, the protein function in autophagy regulation is also correlated with interactions with other specific partners.

It is well known that autophagy is promoted by diverse cellular stresses including oxidative stress, metabolic stress under nutrient deprivation, endoplasmic reticulum (ER) stress, and inflammation.

### 3.1. Interplay p62-Nrf2/Keap1

**Figure 2:**
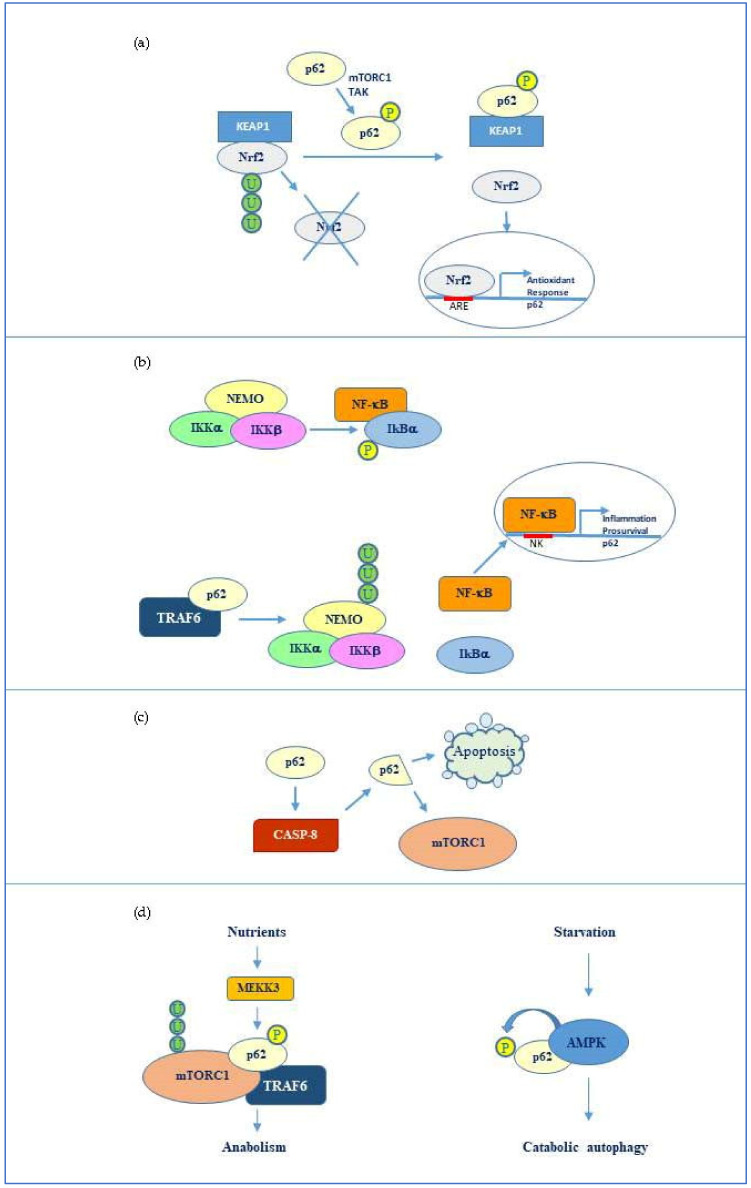
p62 molecular interactors and relative signalling. (**a**) Phosphorylated p62 (Ser349) activates nuclear factor (erythroid-derived 2)-like 2 (Nrf2)-mediated oxidative response and cell survival by sequestering Kelch-like ECH-associated protein 1 (Keap1). p62 expression is then activated by Nrf2 in an amplification loop. (**b**) p62 promotes NF-kappa-B essential modulator (NEMO) ubiquitination via TRAF6 favouring NF-κB activation and consequent transcription of genes involved in inflammation and pro-survival response. NF-κB also induces the same p62 expression. (**c**) p62 stimulates caspase-8 which in turns cleaves p62 thus promoting apoptosis or mTORC1-mediated nutrient sensing. (**d**) mTORC1 modulation by p62 via TRAF6 and stimulation of anabolism in the presence of nutrients and AMP kinase mediated p62 phosphorylation during starvation and consequent catabolic autophagy.

Concerning oxidative stress, p62 is known to take part in the Nrf2/Keap1 mediated signalling, an important cytoprotective and antioxidant response mediated by the activation of the transcription factor Nrf2. The levels of Nrf2 are kept low in the cells under normal conditions since the factor is degraded by the ubiquitin proteasome system. This event is promoted by the Nrf2 inhibitor Keap1, which binds Nrf2 and promotes its ubiquitination and proteasome-mediated degradation. Upon oxidative stress, Keap1 undergoes a conformational change that decreases affinity to Nrf2 [50]. Consequently, Nrf2 is released and translocates to the nucleus, where it promotes the expression of antioxidant and pro-survival genes. p62 has been shown to interact, through its KIR domain, with Keap1 at the same binding site for Nrf2 and thus competitively inhibits the Keap1/Nrf2 interaction resulting in Nrf2 activation (Figure 2a). As previously mentioned, binding to Keap1 is favoured by phosphorylation of Ser349 present in p62 KIR domain [45]. Different kinases including mechanistic target of rapamycin complex 1 (mTORC1) and TGF-β activated kinase (TAK1) can phosphorylate p62 in this residue thereby promoting Nrf2 activation. Notably, p62 and Nrf2 have a mutual relationship since Nrf2, which can be activated by p62, can in its turn stimulate SQSTM1 gene transcription [51]. Moreover, p62 binding to Keap1 seems to target Keap1 for inclusion in the autophagosome and consequent degradation by selective autophagy [52]. On the other hand, evidence has been provided that Keap1 can favour p62 ubiquitination in the UBA domain, an event that promotes its degradation when autophagy is completed. These evidence suggest that Nrf2/Keap1 pathway and p62 mediated-autophagy are coupled with each other. This connection is particularly important in cancer development and will be discussed in the next section.

### 3.2. p62 Relationship with NF-κB and Caspase 8

Nuclear factor-κB (NF-κB) represents a family of transcription factors that are fundamental in inflammatory responses and are considered among the most important molecules linking chronic inflammation to both cancer and neurodegenerative diseases [53,54]. Recent evidences support an interesting interplay between p62 and NF-κB mediated signalling, which can be decisive to balance autophagy with classic apoptosis.

P62 can activate NF-κB via its tumor necrosis factor (TNF) receptor associated factor 6 (TRAF6) binding motif (TB), thereby enhancing inflammatory responses mediated by IL-1 or TNF [41] or anti-apoptotic and pro-survival signals [55]. NF-κB, in its turn, can stimulate p62 gene transcription, constituting a feed-forward loop that amplifies its signalling effects [24]. Concerning TRAF6, p62 seems to promote TRAF6-mediated Lys63-linked ubiquitination of NEMO, a regulatory factor of the NF-κB inhibitory complex (IKK), thus promoting NF-κB release and activation [56] (Figure 2b). Oppositely, p62 may also inhibit TRAF6 signalling [57] and promote caspase-3 activation and consequent apoptosis execution [58]. In addition, Young et al. have shown that poly-ubiquitination of p62 favours Fas/CD95 aggregation to induce caspase-dependent apoptosis [59]. In this scenario, the role of p62 in apoptosis appears to be dual since the protein can either block apoptosis favouring autophagy in an initial phase or promote apoptosis switch following autophagy triggering. For instance, p62 has been shown to activate caspase-8 under oroxylin treatment in hepatocellular carcinoma and to be further degraded by the same active caspase. This generated a cleaved p62 form that lost the ability to activate Nrf2, thus promoting oxidative stress and cell death [60]. In another experimental model, Garrido et al. have shown that a cleaved p62 form obtained from proteolytic cleavage by caspase 8, instead of functioning in autophagy or in apoptosis, was selectively involved in nutrient-sensing and cell homeostasis through mTORC1 [61] (Figure 2c).

### 3.3. p62 Interaction with mTORC1

Among p62 interactors, mTORC1 represents another key factor in cell fate regulation. Specifically, mTORC1 is a complex composed of mTOR (the effective kinase subunit) associated with Raptor, a component important for mTORC1 localization and substrate recognition, and mLST8, which promotes mTOR kinase activity. The activity of the whole complex is negatively modulated by PRAS40 and DEPTOR, two inhibitory subunits that are also present in the complex [23]. mTORC1 is a major complex involved in nutrient sensing and represents a crossroad between catabolic and anabolic pathways in the cells [62]. In the presence of nutrients, mTORC1 promotes anabolic processes including protein, lipid, and nucleotide synthesis to favour cell growth, concomitantly inhibiting catabolic pathways such as autophagy. The inhibitory effect of mTORC1 on autophagy is related to phosphorylation of ULK1, an autophagy-initiating kinase, promoted by the complex [63]. Surprisingly, p62, which is clearly involved in autophagy, has been shown to activate mTORC1 [64]. In particular, evidence has been provided that p62 is phosphorylated at Thr269 and Ser272 by MEKK3 in response to amino acid abundance [65]. In this phosphorylated form, p62 recruits the ubiquitin ligase TRAF6 and mTORC1 resulting in Lys63-linked ubiquitination and consequent activation of mTOR (Figure 2d). This most likely represents an inhibitory feedback that p62 promotes with respect to catabolic autophagy under nutrient abundance, coupling this condition to anabolism and cell growth.

Conversely, energy reduction due to nutrient deprivation activates the metabolic sensor AMP kinase which inhibits mTORC1 directly by phosphorylation and indirectly by stimulation of its inhibitor mTORC negative regulator (TSC) [66]. AMP kinase has been shown to phosphorylate p62 in Ser294 following insulin withdrawal in hippocampal neural stem cells, thus promoting autophagy in this model [67] (Figure 2d). Implication of p62 in cell metabolism regulation together with its role in balancing cell death and survival open a new perspective to understand the molecular basis of both cancer and neurodegenerative diseases.

## 4. *OncoJanus* Role of p62

The versatile properties of p62 and diversified roles deriving from the broad spectrum of interactors make the protein a double-edged sword in cancer. A number of papers support an oncogenic role of p62 in a wide variety of human cancers [21]. However, recent findings also suggest that the protein may behave oppositely counteracting tumor development and exerting a pro-apoptotic function [20]. This paragraph aims to critically revise the most recent literature in this regard and discuss the *oncoJanus* role of p62.

### 4.1. Pro-Tumor Role of p62

Accumulation of p62 has been found in several forms of tumors including hepatocarcinoma, breast, lung, gastric, colon, and ovarian cancers [68,69,70,71,72]. Increasing evidence demonstrates that abnormal expression of p62 is associated with malignancy in most cases. For instance, an amplified p62 copy number on chromosome 5q has been found in renal cancer and overexpression of the SQSTM1 gene was identified in many other tumors [23,73]. Notably, p62 knockout or knockdown have been shown to abrogate tumor growth in different cancer models [74,75]. In such a way, p62 may be considered as an oncogene.

From a molecular point of view, given that p62 abundance may favour pro-survival tumor-associated autophagy, increased activity of p62 was also correlated with increased phosphorylation. In particular, the phosphorylated form of p62 in Ser349 leads to persistent activation of Nrf2 signalling promoting cell survival and consequent tumor growth. Interestingly, Ichimura et al. have identified a specific inhibitor for the Keap1/phospho-p62 (Ser349) interaction, the acetonyl naphthalene derivative K67 [45]. These authors showed that treatment of hepatocarcinoma cells with K67 suppressed proliferation and reduced tumor resistance to cisplatin or sorafenib [76].

p62/Nrf2 signalling can be also negatively regulated by the tumor suppressor PCDC4 (programmed cell death 4). In this regard, evidence has been provided that induction of PDCD4 overexpression in lung cancer increased Keap1 levels and reduced the activity of the p62/Nrf2 pathway, thereby inhibiting tumorigenesis [77]. In addition, other authors have shown that p62 knockdown in hepatoma cells upregulated PCDC4 levels and demonstrated that p62, PCDC4 and LC3 co-localized in particles to promote PCDC4 autophagic degradation [78].

Other molecular events that may explain p62 oncogenic potential are correlated with NF-κB or mTORC1 activation. Recently, Sanchez Lopez et al. have found NF-κB-driven p62 expression in chronic lymphocytic leukemia (CLL) correlates with both Nrf2 and mTORC1 activation [79].

Of relevance is the finding of these authors that CLL cells expressing on the surface high ROR1, an onco-embryonic orphan receptor, display increased p62/Nrf2-mediated antioxidant response and consequent resistance to pro-oxidative drugs such as venetoclax. Notably, they propose a possible targeted therapy for this neoplasia consisting in administration of a drug that is selectively activated by antioxidant enzymes induced by p62/Nrf2 signalling.

As previously discussed, mTORC1 is strictly associated to anabolism and cell growth, therefore cancer cells are highly benefited by increased activity of this factor. Activation of mTORC1 related to p62 upregulation was found, for instance, in hepatocellular carcinoma [68]. Moreover, p62 has been recently shown to play a key role in cellular redox homeostasis by keeping mitochondrial integrity and glutathione levels necessary to support mTORC1 hyperactivation [73]. Notably, Tamura et al. have shown that combination of a dual PI3K/mTOR inhibitor with p62 knockdown targeted human bladder cancer either in vitro or in vivo [80]. More recently, Xu et al. provided evidence that p62 was enriched in small cell lung cancer spheroids with cancer stem-like properties and that p62 knockdown sensitized these cells to cisplatin [81].

### 4.2. Antitumor Role of p62

The other side of the coin is that p62 can exert a protective function against tumorigenesis. For instance, p62 levels were found to be reduced in the stroma of several tumors and loss of p62 in the tumor microenvironment resulted in increased tumorigenesis [82]. In particular, the same authors showed that inactivation of mTORC1 in p62-deficient stromal fibroblasts caused a metabolic reprogramming that increased stromal IL-6 production, which promoted inflammation and tumor growth. Therefore, in this context, p62 seemed to behave as an anti-inflammatory and tumor suppressor.

Metabolic reprogramming is a hallmark of tumor cells. A fascinating study of how p62 is implicated in the communication between metabolic tissues and cancer cells is provided Yuang et al. Specifically, these authors propose p62 as a potential metabolic tumor suppressor and describe the symbiotic collaboration between adipose tissue and tumor cells that profoundly affects cancer metabolic fitness [83]. In addition, p62-deficient tumor stroma has been shown to produce asparagine that is released and sustains tumor metabolism in conditions of glutamine deprivation [84].

In the scenario of tumor microenvironment and communication of tumor cells with other cell types, a relevant function of p62 has been identified in tumor surrounding macrophages.

Induction of p62 through NF-κB in macrophages serves to limit the release of cytokines, such as IL-1β and consequent inflammation, which is likely to promote tumor survival and growth [24]. Moreover, p62 exerts a protective function in macrophages since p62-dependent autophagy promotes the clearance of mitochondria that have been altered following inflammasome activation in these cells [85]. It is well known that inflammasome activation in tumor associated macrophages promotes tumorigenesis. These evidences indicate that p62 preserves macrophage homeostasis and reduces their oncogenic potential. Overall, p62 displays a key role in the tumor microenvironment through a fine regulation of the crosstalk between different cell types and cancer cells in the tumor niche.

Intriguingly, p62 has also been shown to exert an anti-tumor function within cancer cells. For instance, at the nuclear level p62 can control DNA damage through inhibition of Histone H2A ubiquitination, thus affecting DNA repair and increasing the sensitivity of tumor cells to radiation [86]. In such a perspective, high levels of p62 can even represent an advantage for cancer therapy.

In accordance, Yan et al. have shown that overexpressed wild-type p62 in ovarian cancer cells significantly increased activation of caspase 8, with consequent apoptosis, and ameliorates the response to cisplatin [20]. Moreover, evidence has been provided that p62 ectopic expression in vivo reverts tumor grade, changes tumor stroma and enhances anticancer immunity [87]. Even more recently, Choi et al. showed increased levels of nuclear p62 upon ascorbic acid treatment in breast cancer cells correlated with endoplasmic reticulum stress and cell death [88]. Although the precise role of nuclear p62 remains to be clarified, it is relevant that this localization seems to be related with an anti-tumor function. In this regard, Liu et al. showed that low nuclear level of p62 was associated with aggressive clinic pathologic features and unfavourable prognosis of oral squamous cell carcinoma [89].

Intriguingly, a p62 encoding plasmid named Elenagen was used in association with standard chemotherapy to treat a chemoresistant triple-negative breast cancer patient with significant improvement in the response and increase in progression-free survival [90].

Taken together, all these findings strongly support the dual role of p62 in cancer. If on the one hand we consider p62 as an oncogene because of its overexpression in many tumors, on the other hand this condition may be strategically used against cancer to improve the therapeutic efficacy. Of relevance is the role of p62 in cells of the tumor microenvironment, which sustains its tumor suppressor properties. Elucidating the interaction mechanisms of tumor cells with other cell types within the tumor niche will definitely shed light on the specific p62 function in tumor development and progression. From a general perspective, we have to consider that the behaviour of the factor can be cell specific and may depend on a number of variables and interaction networks. Pro-tumor and anti-tumor activities of p62 are summarized in Table 1.

## 5. *NeuroJanus* Role of p62

Given the role of p62 in selective autophagy and implications in regulating the balance between cell death and survival, it is not surprising that this multifunctional protein is also involved in the pathogenesis of neurodegenerative diseases. Due to its functional plasticity, p62 serves indeed as a signalling hub for multiple pathways implicated in neurodegeneration. p62 may be neuroprotective since it promotes pro-survival autophagy against various types of neuronal stress or it may be neurotoxic when overexpressed or deregulated. Moreover, many evidences indicate that aberrant p62 is found in association with specific aggregates that are typical of different neurodegenerative diseases [22,99]. This paragraph summarizes and discussed the most recent findings about the opposite roles of p62 in neurodegeneration, focusing on three major neurodegenerative disorders: Alzheimer disease (AD), Parkinson disease (PD), and Amyotrophic lateral sclerosis (ALS).

### 5.1. p62 in Alzheimer Disease

AD is one of the most widespread neurodegenerative diseases and the most common cause of dementia. It is a progressive disorder that causes severe cognitive dysfunctions and decline of memory. The molecular basis of AD is related with β-amyloid (Aβ) plaques accumulation in the brain and hyperphosphorylated tau-containing neurofibrillary tangles within the neurons [100].

Low expression of p62 has been found in many forms of AD and p62 loss of function has been shown to result in Aβ accumulation, tau hyperphosphorylation, and consequent neurodegeneration [101,102].

Interestingly, overexpression of p62 was able to rescue cognitive deficit in transgenic AD mice models, increasing neuronal pro-survival autophagy and affecting Aβ level and amyloid plaque formation. The protective role of p62 is most likely correlated with its capability of promoting the degradation of misfolded protein through the autophagy pathway. Moreover, p62 has been shown to inhibit Aβ-induced neuronal death through TRAF6-mediated ubiquitination of p75 neurotrophin receptor, which is known to bind Aβ and transduce death signals [103].

Deregulation of p62 in AD may also depend on increased p62 phosphorylation. In particular, as previously discussed, increased phosphorylation of Ser349 in KIR domain of p62 is related with Nrf2 activation. Tanji et al. revealed that the ratio of phosphorylated (Ser349) p62 to total p62 was considerably increased in the brains of AD patients compared to controls [104]. The role of Nrf2 in Alzheimer disease has been well described in a comprehensive review by Fão et al. [105].

The discussion above sustains that functional p62 level is decreased in AD patients, causing autophagy failure. However, increased autophagy can occur during Alzheimer development and its deregulation may cause neuronal damage [106]. For instance, in some cases, mitophagy, the selective autophagic degradation of mitochondria involving p62, was found excessively intensified in neurons leading to synaptic deterioration and axon degeneration [99]. In this regard, Wang et al. have recently shown that Aβ promotes the accumulation of mitochondrial p62, which was associated with impaired mitophagy correlated with neuronal cell death. In such a way, p62 seems to appear neurotoxic. Restoration of Aβ-induced mitochondrial dysfunction and impaired mitophagy was obtained by overexpressing parkin [107], an ubiquitin ligase that will be described in the next subsection. Moreover, in this connection, autophagy inhibition has been proposed as a tool to attenuate Aβ toxicity and neurodegeneration in Alzheimer transgenic mice models [108].

### 5.2. p62 in Parkinson Disease

PD represents the second most common neurodegenerative disease associated with age, following Alzheimer [109]. Additionally, in this case, it is a progressive disorder in the central nervous system, but it is particularly connected with movement affection since it predominantly compromises dopaminergic neurons involved in motor control. Tremors are indeed the most common signs of early stage PD, then muscle rigidity, slowed movements, and walking difficulty progressively occur. Mental and cognitive problems appear in the advanced stages of PD. The pathogenesis of PD is connected with both genetic alterations, including mutations in PARK genes, and environmental and epigenetic factors. One of the principle pathological factors of PD is the intracellular accumulation of Lewy bodies, consisting of aggregated proteins including α-synuclein, parkin, and ubiquitinated proteins [110].

p62, together with parkin, has an important role in the prevention of dopaminergic neurodegeneration. For instance, loss of p62-dependent autophagy in neurons was associated with Lewy pathology and motor dysfunction in aged mice [111]. In addition, p62-knockdown experiments demonstrated that p62 is required for autophagic clearance of α-synuclein inclusions [112]. Notably, evidence has been provided that autophagy failure can lead to p62 association with α-synuclein accumulation into Lewy bodies. Moreover, it has been shown that aberrant expression of p62 could enhance the amount of α-synuclein in the pathological inclusions [113]. Therefore, a double role of p62 also appears in neurodegeneration. In this context, an important function is exerted by parkin, the product of PARK2 gene, which serves a protective function in dopaminergic neurons.

Being an E3 ubiquitin ligase, parkin has been shown to ubiquitinate p62, thus promoting its proteasomal degradation [114]. The same authors found p62 accumulation and aggregation in dopamingeric neuronal cells of parkin deficient mice. Through the control of p62 levels, parkin, together with the mitochondrial factor pink1, is also associated with p62-dependent mitophagy. In normal cells, dysfunctional mitochondria accumulate pink1 in the outer mitochondrial membrane, with consequent recruitment from cytosol to damaged mitochondria and phosphorylation of parkin. In this form, parkin ubiquitinates mitochondrial substrates that are then recognized by p62 to undergo mitophagy.

Deregulation of pink1/parkin/p62 pathway has been associated to increased vulnerability of neuronal cells toward PD development [114]. In particular, parkin loss of function results in p62 accumulation and association to Lewy bodies.

Overall, if on one hand mitophagy failure leads to p62 accumulation and association with α-synuclein and PD pathogenesis, on the other hand, p62 is fundamental together with parkin and pink1 to maintain neuronal homeostasis and survival.

### 5.3. p62 in Amyotrophic Lateral Sclerosis

Much evidence indicates that p62 is also involved in ALS, a progressive and fatal neurodegenerative disease, which is caused by the gradual depletion of motor neurons in either the cerebral cortex, the brain stem, or the spinal cord. The consequence is an impairment of voluntary muscle movements that leads to paralysis, respiratory arrest, and death. ALS can be familial or sporadic, although the distinction between the two forms can be difficult to evaluate [115]. The pathogenesis is highly correlated with genetic factors, but environmental exposure can also contribute. One common aspect of different ALS forms is the aggregation of insoluble proteins within cells. In particular, intrinsically disordered proteins such as DNA-binding protein 43 (TDP-43) and fused in sarcoma (FUS) are frequently associated to the pathogenic aggregates within motor neurons of ALS patients [116]. A minor percentage of patients (about 20%) have accumulations of superoxide dismutase 1 (SOD1), a key antioxidant enzyme involved in toxic superoxide radicals disposal within the cell [117]. Accumulation of such proteins in amorphous aggregates is due to genetic mutations in their encoding genes or aberrant expression of their inactive forms. Most of these inclusions have been shown to be ubiquitin-immunoreactive and in many cases they contain p62.

It is interesting to note that p62 mutations have been found in a large cohort of both familial and sporadic ALS patients [118,119,120]. These result in accumulation of p62 in the protein aggregates within the motor neurons. Gal et al. showed that accumulation of p62 can enhance the formation of polyubiquitinated proteins and mutant SOD1 aggregates in the spinal cord in a mouse model of ALS [121]. They also provided evidence that p62 can directly bind to a mutated form of SOD1, suggesting that p62 can target mutant SOD1 to selective autophagy in an ubiquitin-dependent mechanism. Autophagy impairment or p62 loss of function may therefore contribute in ALS pathogenesis [122]. Moreover, functional p62, through stimulation of Nrf2- mediated antioxidant response, also serves a protective function against oxidative stress related with SOD1 mutations [123].

More recent findings indicate that p62 can also interact with both TDP43 and FUS, the main components of stress granules in ALS affected motor neurons. In this regard, evidence has been provided that p62 contributes to elimination of stress granules by autophagy [124], thus serving a neuroprotective function.

However, Lee et al. found that functional modulation of p62 can switch toward neurodegeneration. In particular, they showed that p62 phosphorylation at Ser403 increased upon TDP43 overexpression in neuronal cells and this was correlated with accumulation of insoluble poly-ubiquitinated proteins and neurotoxicity [125]. This evidence, together with the observation that p62 can be mutated or functionally altered in ALS patients, confirms that p62 can either suppress or promote neurodegeneration depending on particular circumstances, cell context, and interactive networks. Neurotoxic and neuroprotective roles of p62 are summarized in Table 2.

## 6. Conclusions

Biochemical complexity is a hallmark of multi-factorial diseases, including cancer and neurodegenerative disorders. In a wide and intricate molecular scenario, it is not easy to strictly classify a factor as a positive or negative regulator in the pathogenesis and disease development.

P62 perfectly fits the feature of a double-faced protein depending on cell context, interacting networks, expression levels, and regulative post-translational modifications.

If we can consider p62 a “benefactor” when it contributes to protect the cells from tumorigenesis or neurodegeneration, we have to consider it a “maleficent” when it is de-regulated or overexpressed thus causing the opposite effects. Discriminating such behaviour becomes important to understand the molecular basis of the above-mentioned diseases and to perform appropriate targeted therapies. For instance, future research could be focused on pharmacological tools to target p62 accumulation in particular tumor types or in those neuron-specific aggregates contributing to neurodegeneration. On the other hand, stimulation of p62-mediated autophagy or p62/Nrf2-mediated antioxidant response may result as useful to counteract neurodegeneration or to selectively target tumor cells by autophagic cell death and redox homeostasis control.

## Figures and Tables

**Table 1:** Highlights of research findings of p62double role in cancer.

***p62 pro-tumor activity***			
**Tumor Type**	**Supposed Role**	**Identified Intermediate**	**References**
**Lung cancer**	- Promotion of cisplatin resistance - Resistance to Autophagic Cell Death - Promotion of tumorigenesis	Positive NEDD9 regulation Increased Nrf2/ARE activity and ROS production Increased Nrf2 Signalling and Upregulating Keap1	[81] [91] [77]
**Breast cancer**	- Association with invasive phenotypes - Tumour growth and progression, drug resistance. - Enhancement of breast cancer stem-like properties	Vimentin upregulation CD44-mediated Nrf2 activation MYC mRNA stabilization	[92] [69] [93]
**Gastric cancer**	- Correlation with lymph node metastasis, vessel invasion and hepatic metastasis - High cytoplasmic and low nuclear p62 levels associated with poor prognosis	Autophagy promotion Not determined	[71] [94]
**Colon cancer**	- Independent risk factor for poor prognosis	Not determined	[72]
**Ovarian carcinoma**	- Involvement in cisplatin resistance	NF-κB activation and Lys63-linked RIP1 ubiquitination	[95]
**Bladder Cancer Cells**	- Promotion of Cell Growth - Resistance to apoptotic cell death	Keap1/Nrf2-dependent antioxidant response activation Resistance to PI3K/mTOR inhibitor effect.	[75] [80]
**Nasopharyngeal Carcinoma Cells**	- Induction of pro-survival autophagy, proliferation, migration and invasion	Stimulation of ERK Signaling Pathway	[96]
**Chronic lymphocytic leukemia**	- Promotion of cell survival	Enhancement of mTORC1 signalling and activation of Nrf2	[79]
**Hepatocellular carcinoma**	- Protection from oxidative stress-induced death - Tolerance to anti-cancer drugs, metabolic reprogramming and promotion of malignancy	Activation of Nrf2 and mTORC1, and c-Myc induction Nrf2 activation	[68] [97]
**Renal carcinoma**	- Stimulation of TSC2-driven tumorigenesis	Interaction with mTORC1 complex	[73]
***p62 anti-tumor activity***			
**Tumor Type**	**Supposed Role**	**Identified Intermediate**	**References**
**Prostate cancer**	- p62 loss in the tumor microenvironment and increased inflammation and tumorigenesis - Metabolic reprogramming of tumor associated stroma lacking p62 and tumor growth	Regulation of mTORC1/c-Myc pathway Upregulation of stromal ATF4 by p62 deficiency	[82] [84]
**Ovarian cancer**	- Autophagic flux blockage	Caspase-8 Activation	[20]
**Cervical cancer**	- Sensitization to radiation by nuclear p62	Inhibition of Histone H2A ubiquitination	[86]
**Colorectal cancer**	- Sensitization to photodynamic therapy - Induction of autophagic cell death	Activation of autophagic cell death pathway	[14,98]
**Breast cancer**	- Mediation of ascorbic acid anti-proliferative effect. - Chemoresistance attenuation by p62 encoding plasmid	Induction of IRE/JNK/CHOP -Related ER Stress Not determined	[88] [90]
**Mammary carcinoma**	- Reversion of tumor grade and anticancer immunity stimulation by ectopic expression of p62	Increase in intra-tumor T cells	[87]
**Melanoma**	- Tumor growth reversion by p62 encoding plasmid	Not determined	[87]
**Oral squamous cell carcinoma**	- Association of low nuclear p62 expression with aggressive clinicopathologic features	Not determined	[89]

**Table 2:** Highlights of research findings of p62 double role in neurodegeneration.

Neurodegenerative Disease	p62 Status	Supposed Mechanism or Identified Interactor	References
***p62 Neurotoxic Function***			
**Alzheimer**	- Increased p62 (Ser349) phosphorylation - Increased levels	Nrf2 signalling aberrant increase Excessive mitophagy	[104] [99]
**Parkinson**	- Aberrant expression - Accumulation and aggregation in dopamingeric neurons	Increased α-synuclein in pathological inclusions Parkin deficiency	[113] [114]
**Lateral Amyotrophic Sclerosis**	- Mutation and accumulation - TDP43 overexpression dependent p62 (Ser403) phosphorylation	Protein aggregates in motor neurons Poly-ubiquitinated protein accumulation	[120] [118,125]
***p62 neuroprotective function***			
**Alzheimer**	- Low expression or loss of function - Induced expression of p62	Autophagy failure, misfolded protein aggregation, Aβ accumulation, Tau hyperphsphorylation Pro-survival autophagy, Aβ accumulation and toxicity TRAF6-mediated p75 ubiquitination	[22,99] [101,102,103,108]
**Parkinson**	- Normal levels - Loss of p62	Autophagic clearance of α-Synuclein inclusions Parkin/Pink1-mediated mitophagy Lewy pathology and motor dysfunction	[112] [114] [111]
**Amyotrophic Lateral Sclerosis**	- Normal levels	Targeting mutant SOD1 to selective autophagy. Stimulation of Nrf2-mediated antioxidant response Autophagic elimination of TDP43/FUS positive stress granules	[121] [123,124] [125]
